# Identifying and Monitoring the Daily Routine of Seniors Living at Home

**DOI:** 10.3390/s22030992

**Published:** 2022-01-27

**Authors:** Viorica Rozina Chifu, Cristina Bianca Pop, David Demjen, Radu Socaci, Daniel Todea, Marcel Antal, Tudor Cioara, Ionut Anghel, Claudia Antal

**Affiliations:** 1 Computer Science Department, Technical University of Cluj-Napoca, Memorandumului 28, 400114 Cluj-Napoca, Romania; viorica.chifu@cs.utcluj.ro (V.R.C.); daniel.todea@cs.utcluj.ro (D.T.); marcel.antal@cs.utcluj.ro (M.A.); tudor.cioara@cs.utcluj.ro (T.C.); ionut.anghel@cs.utcluj.ro (I.A.); claudia.pop@cs.utcluj.ro (C.A.); 2 Department of Informatics, Technical University of Munich, Boltzmannstr. 3, 85748 Garching, Germany; david.demjen@tum.de; 3 Mobile Clients Team, Prime Video, Amazon, 1 Principal Place, Worship St, London EC2A 2FA, UK; rsocaci@amazon.co.uk

**Keywords:** daily routine, activities of daily living, Beacons, Markov model, entropy rate and cosine functions, deviations from routines

## Abstract

As the population in the Western world is rapidly aging, the remote monitoring solutions integrated into the living environment of seniors have the potential to reduce the care burden helping them to self-manage problems associated with old age. The daily routine is considered a useful tool for addressing age-related problems having additional benefits for seniors like reduced stress and anxiety, increased feeling of safety and security. In this paper, we propose a solution for identifying the daily routines of seniors using the monitored activities of daily living and for inferring deviations from the routines that may require caregivers’ interventions. A Markov model-based method is defined to identify the daily routines, while entropy rate and cosine functions are used to measure and assess the similarity between the daily monitored activities in a day and the inferred routine. A distributed monitoring system was developed that uses Beacons and trilateration techniques for monitoring the activities of older adults. The results are promising, the proposed techniques can identify the daily routines with confidence concerning the activity duration of 0.98 and the sequence of activities in the interval of [0.0794, 0.0829]. Regarding deviation identification, our method obtains 0.88 as the best sensitivity value with an average precision of 0.95.

## 1. Introduction

In Europe, it is estimated that the number of older adults will continue to increase from 90.5 million at the start of 2019 to reach 129.8 million by 2050 [1]. At the same time, the number of formal and informal caregivers will not grow to match the increasing need, resulting in higher care costs and a decrease in quality of life [2]. The remote monitoring and assessment solutions integrated into the living and working environment of seniors have the potential to reduce the care burden, helping them to self-manage problems associated with old age. Moreover, older adults’ chronic condition is an important factor that should be considered, being complicated by additional risk factors such as deficits in activities of daily living, social situations exposing them to isolation and lack of support, cognitive decline and emotional anxiety [3]. Poorly managed care often inadequately considers the seniors’ health decline due to the lack of remote objective monitoring and does not address promptly potential problems.

Studies show that slowing the cognitive decline and maintaining independent functioning in conducting the activities of daily living (ADL) are important goals in supporting the care and wellbeing of older adults [4]. Using ambient assistive technologies in older adults’ homes is a promising solution for better managing their condition, helping them to live independently with as little support as possible from caregivers. In this context, the daily routine is considered a useful tool for addressing cognitive decline and self-management of chronic conditions having additional benefits like reduced stress and anxiety, increased feeling of safety and security. The use of IoT sensors and statistical and computational intelligence methods is promising as they allow formal and informal caregivers to establish and monitor the daily routines of older adults in an objective manner and to detect potential deviations requiring intervention. Following a daily routine induces a state of calm and comfort, for older adults reducing the level of anxiety and stress, and can increase the likelihood of being transferred into the long-term memory, this being very important in case of cognitive decline. By performing regular activities from the daily routine, their self-esteem and confidence may increase, as they are able to perform activities independently.

Equally important is the continuous monitoring of daily routine and any deviations from it. This can contribute to the identification of decreasing functionality in activities of daily living as well as of cognitive decline [5]. The traditional screening methods for assessing the capacity to conduct daily activities are usually based on self-reporting and they lack contextual information, not allowing for wider adoption. The use of innovative non-invasive monitoring solutions may allow the identification of sudden or gradual deviations from the baseline routines, allowing the setup of personalized intervention processes prolonging the autonomy and well-being of older adults. Such solutions could also improve the quality of life and could help older adults to live more years in a meaningful and dignified manner. Unfortunately, even when the older adult with noticeable decline symptoms visits the healthcare professional for an assessment, research shows that, in many cases, such deviations are difficult to identify due to a lack of information.

In this paper, we propose a solution for identifying the daily routines of seniors using the monitored activities of daily living and for inferring deviations from the baseline routines that may require professional or informal caregiver interventions. The paper’s novel contributions are the following:

- Markov model-based method for identifying the daily routines of older adults considering the daily living activity probability transitions and activity length.
- Technique for identifying relevant deviations from daily routines using entropy rate and cosine functions to measure and assess the similarity between the sequence of activities registered in a specific day and the baseline routine.
- Distributed system for testing and evaluation of the proposed methods which uses Beacons and trilateration techniques for monitoring the activities of the daily living of older adults.

The paper is structured as follows: Section 2 reviews some of the existing approaches in the field of daily routine assessment and anomalous behavior detection, Section 3 presents the daily routine assessment method, Section 4 presents the solution for deviation from baseline routine identification, Section 5 presents validation and the experimental results, while Section 6 concludes the paper.

## 2. Related Work

In the studied literature, the existing approaches for behavior anomaly detection differ based on the number of daily activities considered, strategies used to detect normal/abnormal behaviors and the features considered as relevant in the process [6,7,8]. The simplest type of anomaly is the punctual anomaly. In this case, each daily activity is considered independent, and the anomaly identification does not consider the potential relations with other activities. Several approaches aim to identify such anomalies in the case of elders with mild cognitive impairments living in smart homes. They use rule-based induction [9], statistical and knowledge-based methods [10,11] or combine clustering algorithms with recurrent neural networks [12].

A prerequisite in this case is the accurate detection of daily activities. In [13] a two-layer Hidden Markov Model (HMM) is used to learn and recognize basic activities. In the first layer, location data from sensors is used to predict the activity class and in the second layer the prediction is refined for activities under the same class. It obtains better accuracy than approaches that use Naive Bayes or Conditional Random Field, but it does not consider the model integration with sensor-based monitoring infrastructure. In [14] a hybrid approach for recognizing ADL is proposed using smartphone sensors combined with ambient ones. A coupled Hidden Markov model is used to model the temporal evolution of each person’s activity, while specific spatiotemporal constraints are used to limit the viable state space of activities. The approach features good results; however, it is difficult to use with older adults, as they will have to carry their smartphone with them all the time. A deep-learning framework to recognize complex ADL leveraging onto activity state representation while considering motion and environment sensor data is proposed in [15]. The results obtained are better compared with HMM and LSTM (Long Short-Term Memory) approaches, but it does not feature any anomaly detection. In [16] the authors propose a solution, which is the use of wearable sensors, convolutional and LSTM recurrent units to learn ADL features and temporal dependencies. It offers better accuracy compared with Convolutional Neural Networks (CNNs) based approaches, but limited results have been provided considering monitored data.

In the case of collective anomalies, a group of activities is analyzed together to identify whether the group or sequence is normal or abnormal. Authors of [17] uses data collected from wearable sensors to detect anomalies in elders’ behavior. The authors define a probabilistic model based on several parameters such as location, duration, start time and activities sequences. In [18] signals collected from an accelerometer are combined with ECG signals to detect users’ behavioral anomalies, while in [19] a supervised machine learning algorithm to classify anomalous sequences of activities is proposed. In the classification of contextual anomalies, daily activities are analyzed considering contextual features such as weekday or weekend, medication, etc. Solutions are proposed for real-time anomaly detection, with the contextual features being used for pruning the identified anomalies [20,21]. The daily patterns are detected with machine learning techniques and heuristics are employed to select relevant contextual features and fine-tune the learning parameters [22]. The spatial, temporal and contextual features are used to detect other types of anomalies such as incomplete activities, confusion in performing the activities, repeating activities or disruption of sleep [10,23,24]. Regardless of the anomaly type, two classes of strategies can be used for abnormal behavior detection. The profiling strategy supposes learning a model showing normal behavior [11,25]. The model is then used to detect anomalies in the new incoming data. The behavior is considered abnormal if there is a deviation from the learned model. In the case of the discriminating strategy, the abnormal behavior is included in the training data and the learned model is used then on incoming data [24,26].

The algorithms used for implementing the abnormal behavior detection strategies are either statistical or based on machine learning techniques. In the first case, statistical methods are used to detect abnormal behaviors. In [27], Hidden Markov Models (HMMs) are constructed from monitored data to predict the changes that could appear in the health status of older adults. The solution presented in [11] detects abnormal behavior in the case of elders using Bayesian statistics. Three types of likelihoods are considered: the sensor activation, the sensor sequence firing, and the event duration. In [28] a transition probability-based matrix which models the daily activity inside a room and room-to-room transitions is presented. A transition probability matrix is defined and used to model the daily mobility behavior of a person. Authors of [29] use a probabilistic Spatio-temporal statistical model to identify the daily behavior of an elder, and a cross-entropy measure to determine significant deviations. The daily routine of an elder is modeled as a collection of behavioral places located arbitrarily in a generic space [30]. Virtual pheromones are used to build images of the distribution maps which describe the evolution in space and time of the interactions between the elder and the environment. The deviations from the daily routine are detected by applying statistical analysis on top.

Computational intelligence methods such as supervised, semi-supervised and unsupervised learning have been used in the literature to detect normal and abnormal behavior patterns. In [31] Random Forest algorithms are used to create clusters of human behavior patterns, while agglomerative clustering is used to reveal data clusters into a 2D space. In [32], the kernel K-means algorithm is combined with a novel nominal matrix factorization method to detect the daily living routine of an elder. A behavior-aware flow graph is built to represent the trajectory data, then a kernel k-means algorithm is used to identify sub-flows representing behavioral patterns, and finally, a nominal matrix factorization method is used to identify the daily routine. In [33] the k-means clustering algorithm is applied to extract the daily behavior model of a person, and then the model and a cross-entropy measure are used to detect anomalies. Neural networks are used for behavioral anomaly detection [24]. Vanilla Recurrent Neural Networks (VRNNs), Long Short Term RNNs (LSTMs) and Gated Recurrent Unit RNNs (GRUs) are used in the case of elders with dementia to recognize daily life activities and to learn daily life behavioral routines. Authors of [24] propose a method based on Convolutional Neural Networks (CNNs) to identify abnormal behaviors such as repetitive activities, sleeping problems and confusion in performing an activity, etc. In [26] Long Short-Term Memory (LSTM), Convolutional Neural Network (CNN), CNN-LSTM and Autoencoder-CNN-LSTM are used to identify and predict the abnormal behavior of elders. The four networks have been applied on two public data sets, namely SIMADL Dataset [34] and MobiAct Dataset [35], and the experimental results demonstrate that hybridization of CNN with LSTM provides the best results in the case of detecting temporal and spatial abnormal behavior. In [36] an unsupervised approach for learning the ADL routine of elders living alone and detecting deviations from it is proposed. The daily living activities are identified by correlating the elder’s location in the house with the location’s power consumption. The approach deals with identifying the activities of daily living, modeling the basic routine of the elder and detecting the deviations from the routine in new sensor data using a fuzzy inference system to detect deviations from the routine. A Probabilistic Neural Network for activity recognition and an H_2_O autoencoder to identify anomalies about activity duration and the number of subevents are combined in [25]. In [37] the daily routine is sketched in collaboration with the monitored person, who is asked to describe the activities carried out daily. Based on the identified routine, a score is calculated that reflects how well an activity fits with the daily routine. In [7] the normal behavior of a person is defined as a sequence of four activities (sleeping, eating, taking a shower and leaving home), which are performed at specific times of the day. For detecting the behavior model, an unsupervised approach based on the DBSCAN algorithm is applied and the deviations are detected by computing a similarity score between the current behavior of the elder and her/his normal behavioral pattern. Other methods are based on a graph or task models. In [38], a graph that represents the sequence of performed activities and the duration corresponding to each activity for a specific participant is built and used to detect abnormal behavioral anomalies. In [39] a method for detecting behavioral changes in the daily routine of a person is defined by comparing activity curves that model the daily activity routines of a person between different points of time. In [40] the authors propose a method that compares the elder’s expected behavior with the elder’s actual behavior registered as a sequence of events unfolded in the current context. The elder expected behavior is represented as a task model which consists of sequences of tasks performed by an elder in a day (i.e., wake up, go to the bathroom, take medicine without food, prepare breakfast, take another medicine).

This paper builds upon the existing state of the art by proposing a solution for identifying the daily routines of older adults considering the length of the monitored activities and transition probabilities among activities as relevant features. The daily activity monitoring is done using Beacon technology, which offers an affordable, easy-to-install solution with a high potential of personalization since it may be associated with specific objects and related to activities that are being conducted. For identifying the daily routine, we have proposed a Markov-based model. Since the daily routine of a person is defined as the sequence of activities it is suitable to use a probabilistic model in which the transitions between activities are modeled using probabilities. Existing approaches in the reviewed literature based on probabilistic models such as [11,17] consider a few types of probabilities as parameters in their models, identifying a limited precision of the potential deviations. In our approach, new parameters have been considered to improve the model precision such as (i) the activity length probability used to identify the most likely length for the daily routine, (ii) the probability of conducting an activity is used to identify the most likely activities to be part of the routine and (iii) the probabilities of an activity to be the start or end activity of the routine. For anomaly detection, we used a two steps approach that is based on entropy rate and cosine similarity measure. The entropy rate offers the flexibility to consider the days for which we have only minor fluctuations compared to the daily routine in terms of the sequence of activities performed as normal and those for which there are major fluctuations as abnormal. In this way, we introduce the concept of flexibility in defining the daily routine of a person and the days that follow the daily routine. The cosine similarity measure was used to reduce the false-positive rate that could be generated by using the entropy rate. The days that are classified as being normal thus without significant deviation using the entropy metric are then further assessed using the cosine similarity metric to improve the precision of deviation detection. As a result, the precision of our approach is better, as shown in the experimental evaluation section of this paper.

## 3. Daily Routine Detection

For baseline evaluation, we used a Markov model considering activity sequencing, (i.e., the order in which they are conducted) and activity transition probabilities. Four types of probabilities were considered.

The first one is the transition probability, $P\left(A_{i},\;A_{j}\right)$, which refers to the probability of activity $A_{j}$ succeeding activity $A_{i}$. To calculate the transition probabilities, we considered the number of times the specific activity transition appears ($A_{i}\to A_{j}$) and divided it by the number of all transitions starting with the same activity $A_{i}$ but not having as successor activity $A_{j}$:

(1) $$P\left(A_{i},\;A_{j}\right)=\frac{\sum A_{i}\to A_{j}}{\sum A_{i}\to A_{k},\;k\neq j}$$

**Figure 1:**
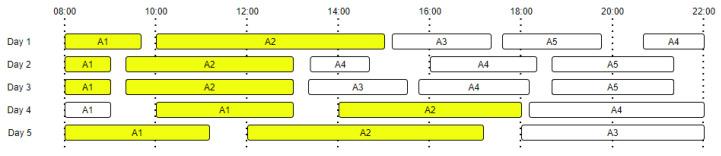
Daily activities and transition probabilities ($A_{1}$ to $A_{2}$ transitions are marked with yellow).

Figure 1 presents an example of daily activity transitions for five days, highlighting all the activity transitions from $A_{1}$ to $A_{2}$. The number of transitions starting from a specific activity is equal with the total number of occurrences of that activity, which means that even if the activity is the last one from a day and has no activity following it, we will still count it in the total number of events. In our approach we have considered daily routines; thus, the last activity of one day does not transition into the first activity of the following day.

The second probability is the occurrence probability of an activity $A_{i}$, $P\left(A_{i}\right)$, which represents the probability with which an activity $A_{i}$ is executed by an older adult. It is computed as:

(2) $$\;P\left(A_{i}\right)=\frac{count\;\left(A_{i}\right)}{\sum_{k=1}^{n}count\;\left(A_{k}\right),\;k\neq j}$$

where $count\left(A_{i}\right)$ represents the number of occurrences of the activity $A_{i}$, $count\left(A_{j}\right)$ represents the number of occurrences of an activity $A_{j}\;$(including $A_{i}$ activity) in the training data set and n represents the total number of activities from the training data set.

The third and fourth probabilities are the probability of activity $A_{i}$ being the first activity of the day ($P_{S}\left(A_{i}\right)$) and the probability of activity $A_{i}$ being the last activity of the day ($P_{E}\left(A_{i}\right)$). They are computed by dividing the number of days starting with activity $A_{i}$ and, respectively, ending with activity $A_{i}$ by the total number of days.

For baseline detection we construct the transition probability matrix (which corresponds to the Markov model) with each position in the matrix, with (i,j) representing the probability $P\left(A_{i},\;A_{j}\right)$ that activity $A_{j}$ follows activity $A_{i}$:

(3) $$MM=\left(\begin{array}{ccc} P\left(A_{1},\;A_{1}\right) & \cdots & P(A_{1},\;A_{n})\\ \cdots & P\left(A_{i},\;A_{i}\right) & \cdots \\ P\left(A_{n},\;A_{1}\right) & \cdots & P\left(A_{n},\;A_{n}\right) \end{array}\right)$$

The baseline is modeled as the most likely sequence of activities that the older adult will do in a specific day:

(4) $$\left(B,\to \;\right)=\;\{A_{i}|\forall \;i=1..d,\;\exists \;A_{i+1}\;such\;that\;A_{i}\to A_{i+1},\;A_{1}\;first\;activity\;of\;the\;day,\;A_{d}\;last\;activity\;of\;the\;day\}$$

The baseline detection algorithm iterates through the whole set of daily living activities and considers for each activity the probabilities to be first or last in the day as well as the transition probabilities. The iteration is started from the most likely activity to be the first one in a specific day (the activity with the highest $P_{S}\left(A_{i}\right)$). For each activity $A_{i}$ we compare the probability that the activity is the last one in the daily sequence, $P_{E}\left(A_{i}\right)$, with all the transition probabilities $P\left(A_{i},\;A_{j}\right)$. If a transition probability higher than $P_{E}\left(A_{i}\right)$ is found, then the activity $A_{j}$ becomes the current activity and $A_{i}$ and $A_{j}$ are added in sequence to the daily baseline. The algorithm stops and returns the baseline when no other activity $A_{z}$ can be found such that $P_{E}\left(A_{i}\right)<P\left(A_{i},\;A_{z}\right)$, where $A_{i}$ is the last activity in the baseline sequence. The EP variable is used to fine tune the importance of the activity in the baseline detection algorithm.

However, when individuals always end their days with the same activity or follow a strict transition activity the algorithm stopping condition might be affected. If the end activity probability is not evenly distributed, the algorithm tends to favor a shorter baseline, stopping whenever the most likely ending activity is reached (i.e., no transition probability is greater than the end probability in this case). Alternatively, if two activities with high transition probability between each other are encountered, the algorithm would tend to bounce from one activity to the next indefinitely, thus favoring longer baselines. To address this issue, we considered another feature, the activity length probability. We determine the most likely length for a baseline using a weighted average of the length of daily activities, and then discount the end probability $P_{E}\left(A_{i}\right)$ with respect to the relative distance between the average length and the length of the baseline which is currently constructed. Algorithm 1 presents the pseudocode for the baseline detection. The closer we get to the median length, the smaller the discount should be, thus we used an inversely proportional mapping function denoted “interpolate”.
**Algorithm 1:** Baseline detection considering both activity sequence and activity length**Inputs:**MM—transition probability matrix, $P_{S}$—set holding each activity probability to be the first one of the day, $P_{E}$—set holding each activity probability to be the last one of the day, EP—end probability weight, $L_{M}$—length median, $LD_{Max}$—length probability weight, P—the transition probability from an activity $A_{i}$ to an activity $A_{j}$; **Outputs:** B—activity sequence representing the baseline. **Begin**
1      B← [];
3      $lastVal\;\leftarrow \;max\left(P_{S}\right);$
4      lastActivity← label(lastVal);
4      prevVal ← null, prevActivity← null;
5      $LD\;\leftarrow \;LD_{Max}$
6      **while**
$\;\exists \;A_{i}\;such\;MM\left[lastActivity,\;A_{i}\right]>P_{E}\left[lastActivity\right]\times EP\;–LD$
**do**
7      append(B, lastActivity);
8      prevVal ← lastVal;
9      prevActivity← lastActivity;
10     $LD\;\leftarrow \;interpolate\left(len\left(B\right),\;\left[0,\;L_{M}],[LD_{Max},\;0\right]\right);$
11     $lastVal\;\leftarrow P_{E}\left[prevActivity\right]\times EP-LD;$
12     **foreach** $A_{i}$
**do**
13     $P\;\leftarrow \;MM\left[prevActivity,\;A_{i}\right]$
14     **if**
lastVal<prevVal×P
**then**
15     lastVal ← prevVal×TP;
16     $lastActivity\;\leftarrow \;A_{i}$
17     **end**
18     **end**
19     **end**
20     **return**
B
**End**

First, the probability of occurrence of each potential baseline length $B_{x},\;P_{L}\left(B_{x}\right)$ is computed and then it is used to determine the median length of the daily baseline $L_{M}$ using a weighted average. Linear interpolation is used to map in an inversely proportional manner the $\left[0,\;L_{M}\right]$ interval to the $\left[LD_{Max},\;0\right]$ interval, where $LD_{Max}$ acts as a weight allowing us to tune the emphasis put by the algorithm on the length of the baseline. The value is used to proportionally discount the end probability based on the current length difference between the median and the baseline. As the baseline’s length approaches the median the discount is reduced. When the median length is reached the discount is set to 0, thus effectively removing any notion of length from the algorithm’s logic. As a result, the length probability parameter ensures the termination logic is not affected by unevenly distributed data. The length discount, LD, will be calculated by interpolating in each step the current length of the baseline in the $\left[0,\;L_{M}\right]$ interval, and then mapping it to the [$LD_{Max}$, 0] interval.

**Figure 2:**
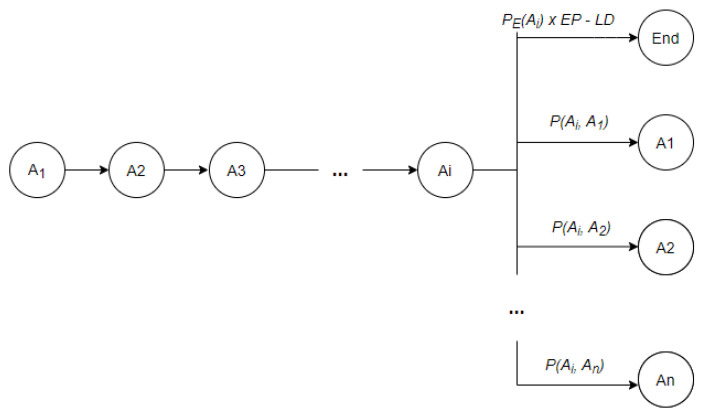
Activity selection in baseline detection process.

Finally, the importance of the baseline length and of the activity end probability can be tuned considering the data at hand using variables LD and EP. Their values can vary between 0 minimum importance and 1 maximum importance concerning the algorithm. Irrespective of the chosen parameters’ configuration the baseline should have the highest Markov product. For example, let us consider that $A_{i}$ is the last activity chosen as part of the baseline sequence at step i. At step i+1 the algorithm has n+1 options to choose from, where n is the number of distinct daily activities (see Figure 2). To yield the maximum Markov product, we need to choose the option with the highest associated probability. The probability to end the sequence with $A_{i}$ as the last activity is calculated as $P_{E}\left(A_{i}\right)$ weighted with EP and discounted by the length discount factor *LD*. If the algorithm cannot find another activity $A_{j}$ to add to the baseline such that $P_{E}\left(A_{i}\right)\times EP-LD<P\left(A_{i},\;A_{j}\right),\;\forall j!=i$ then $A_{i}$ is chosen as the end activity of the baseline and the algorithm’s execution finalizes. Otherwise, if an activity $A_{j}$ satisfying the above condition is found, $A_{j}$ is added to the baseline sequence and the process is repeated with step i+2.

## 4. Deviation Identification

As defined in the previous section the baseline is a sequence of daily living activities executed by an older adult, representing their normal routine. Therefore, to be able to automatically detect anomalies in terms of deviations from the daily routine, a function that associates the sequence of activities registered for a day to a certain similarity value in relation to the baseline is needed. Moreover, an interval of acceptance Δ should be defined in such a way that whenever the value resulting from the mapping falls inside the interval, we accept it as a sequence of activities similar to the daily routine baseline. Alternatively, the day will be flagged as an anomaly or with a deviation from the normal routine.

The entropy rate is a function that has been extensively studied for similarity measures and its pattern detection capabilities [41]. Entropy is usually used as a measure for quantifying information and indicating the degree of randomness in a sequence of activities. Thus, the entropy rate function can be applied to our daily sequence of activities leveraging the already computed transition probability matrix. The following formula is used in similarity computation:

(5) $$\varepsilon =-\underset{i,j}{\sum }P_{ij}\times \;\log\left(P_{ij}\right)$$

where ε is the entropy value and $P_{ij}$ is the transition probabilities from an activity $A_{i}$ to an activity $A_{j}$ in the Markov model associated with the day.

The higher the entropy value, the higher the uncertainty (randomness). If all the daily recorded activity transitions occur with equal probability, then the entropy value reaches its maximum. The entropy rate function value is proven to work well for pattern matching [42,43], but the downside in our case is the fact that it relies only on transition probabilities. For instance, let $P(A_{1},A_{2})=P(A_{2},A_{1})$ for two arbitrary activities $A_{1}$ and $A_{2}$; then, the entropy value used above assigns the same value to both transitions. We could improve the discrimination ability in the case of daily deviation from baseline detection by incorporating the probability of occurrence of each registered activity. In this case the entropy rate is determined as:

(6) $$\xi =-\underset{i,j}{\sum }P_{i}\times P_{ij}\times \;\log\left(P_{ij}\right)$$

The interval of confidence, Δ, is calculated using the entropy rates, using the following formula:

(7) $$\Delta =\xi_{b}\pm \mu \times \sqrt{{\left(\xi_{d}-\xi_{b}\right)}^{2}}$$

where $\xi_{b}$ is the entropy rate of the daily routine baseline, $\xi_{d}$ is the entropy rate of the new registered daily sequence of activities and μ is a confidence index empirically determined considering the size and variance of the data. If the data is scarce and/or has a high deviation, we use a higher confidence index, thus effectively increasing the boundaries of the confidence interval to reduce the false positive rate (days detected erroneously as anomalous). Otherwise, if the data is uniform and in sufficient quantity, we reduce the confidence index for better accuracy. The anomalies are identified as days in which entropy rates of the registered sequence of activities exceed the confidence interval boundaries.

Anyway, considering only the transition probabilities of the daily registered activities in a day is not always sufficient; thus, the duration in time of each activity is also considered. Introducing this second dimension will potentially reduce the false positive rate, therefore improving the accuracy of deviation detection. In this case the daily routine baseline is augmented by associating each activity in the original sequence with its average duration.

(8) $$\left(B,\to \;\right)=\;\{A_{i}|\forall \;i=1..d,\;\exists \;A_{i+1}\;such\;that\;(A_{i},\;avg\;\left(A_{i}\right)\to \left(A_{i+1},\;avg\left(A_{i+1}\right)\right),\;A_{1}\;first\;activity\;of\;the\;day,\;A_{d}\;last\;activity\;of\;the\;day\}$$

The average duration of a specific activity is calculated considering all available historical data by adding the lasting time associated with each activity registration and dividing it by the number of occurrences of that activity. The following formula is used:

(9) $$avg\left(A_{i}\right)=\frac{\sum_{j=1}^{m}t\left(A_{i},j\right)}{m},m=count\left(A_{i}\right)\;$$

where $t\left(A_{i},j\right)$ represents the time duration of the *j*th occurrence of activity *A_i_* and *m* the total number of activity *j* occurrences.

Moreover, in this case comparison measure and a threshold need to be defined and used in the same way in which the confidence interval and the entropy rate were used for the unidimensional deviation detection. The function used to quantify a day similarity in relation to the baseline considering the duration of registered activities is cosine similarity. Cosine similarity is a measure used to quantify the similarity between two or more vectors measuring the “distance” between two vectors in space. The formula below has been used:

(10) $$cosineSim\left(d,\;B\right)=\frac{\sum_{i=1}^{n}t\left(d\left(A_{i}\right)\right)\times t\left(B\left(A_{i}\right)\right)}{\sqrt{\sum_{i=1}^{n}t{\left(B\left(A_{i}\right)\right)}^{2}}\times \sqrt{\sum_{i=1}^{n}t{\left(d\left(A_{i}\right)\right)}^{2}}}$$

where *B* represents the daily routine baseline, *d* is the day for which the sequence of activities is analyzed, $t\left(d\left(A_{i}\right)\right)$ is the duration of the activity $A_{i}$ in the day to be assessed and $t\left(B\left(A_{i}\right)\right)$ is the duration of the activity $A_{i}$ recorded in the baseline. If the two vectors for the current day and for the baseline are 90 degrees apart, the cosine will be 0, indicating maximum discrepancy between the two vectors. Alternatively, if the two vectors are perfectly similar, the angle between them will be 0 degrees, yielding a cosine of 1. Essentially the closer the cosine similarity measurement is to 1, the more similar the two vectors are.

Algorithm 2 shows the pseudocode for the duration-based anomaly detection. The Davg variable stores the average duration for each activity. We assume the functions for computing the average duration, computeAverageDuration, and the one used to compute the cosine similarity of two days, cosineSimilarity, have already been defined.
**Algorithm 2:** Anomaly detectionInputs: TD—testing data set, *NDT*—days that respect the baseline from the training data set, [c_1_,c_2_]—confidence interval, *DSMax*—duration similarity threshold.
Outputs: *AND*—days deviated from baseline, *APD*—days that respect the baseline
1        *ND* ← [];
2        *AND* ← [];
3        *APD* ← [];
4        *Davg* ← *computeAverageDuration*(*NDT*);
5        **foreach**
*day_i_* in TD **do**
6        E ← *computeEntropy*(*day_i_*);
7        **if** c_1_ ≤ E ≤ c_2_
**then**
8          *append*(*ND*, *day_i_*);
9        **else**
10       *append*(*AND*, *day_i_*);
11       **end**
12       **end**
13       **foreach**
*day_i_* in *ND*
**do**
14       *Dt* ← *Davg*
15       **if**
*len***(***Davg***)**! **=**
*len***(***day_i_***) then**
16       *CA* ← *union*(*day_i_*, *Davg*);
17       *day_i_* ← *difference*(*day_i_*, difference(*day_i_*, *CA*));
18       *Dt* ← *difference*(*Davg*, difference(*Davg*, *CA*));
19       **endif**
20       CS ← *computeCosinesSimilarity*(*day_i_*, *Dt*);
21       **if** CS > *DSMax*
**then**
22         *append*(*APD*, *day_i_*);
23       **else**
24         *append*(*AND*, *day_i_*);
25       **end**
26       **end**
27       **return**
*AND*, *APD*

We might have a different length in terms of number of activities for the baseline and the day being compared. In this case, we compute the minimum distance of the two and simply discard the remaining values in the bigger duration sequence. Using this approach, we ensure that a day that has already passed the initial anomaly check (based on ordering and transition probabilities, described in the previous subsection) will not be penalized again. Otherwise, take for example the 0-padding approach (pad the smaller sequence with 0 s), if the day would mimic the baseline up to the second to last entry, with the last activity in the baseline missing, we would have to add a 0 corresponding to the last entry in the duration sequence of the day. When calculating the cosine similarity in this case, the 0 would strongly affect the result of the duration check step. However, the missing activity was already accounted for in the previous step, ordering anomaly detection, and it passed as a day respecting the baseline.

## 5. Evaluation Results

This section describes the system developed for the monitoring and identification of older adults, ADLs, and the results obtained in relation with daily routine detection and deviation identification.

### 5.1. Activity of Daily Living Identification

**Figure 3:**
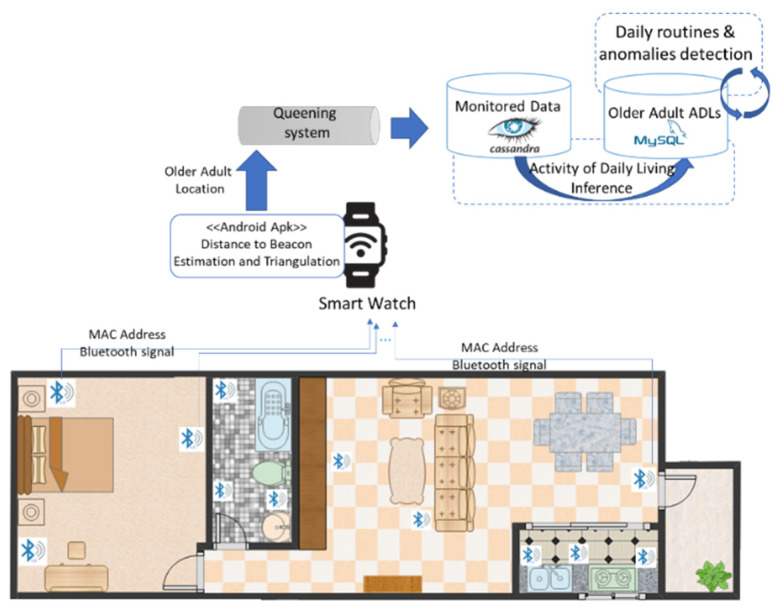
Experimental system for ADL monitoring.

To test our proposed solution, we designed and implemented a distributed system that allows for the identification of daily life activities out of sensors data. The system design is based on stream processing principles for supporting the reliable acquisitions of potential big volumes of data from IoT sensors (see Figure 3).

As sensors, we used Bluetooth Beacons to detect the presence of older adults in different rooms inside their homes and to infer the potential daily activity conducted. The Beacons were configured as shown in Table 1 where the Advertising Interval represents the time between advertising packets that are sent periodically by the Beacon on each advertising channel, txPower is used to adjust a device transmit power level based on the Received Signal Strength Indicator (RSSI) and Cal. power 0 m indicates the level of power received after any possible loss in the environment from the antenna level to the receiver.

In such a Beacon-based monitoring system, data privacy and providing a secured data link between the sensors and the software tools is rather challenging. By default, the Beacons do not send encrypted data and the data can be tampered with. To deal with this type of concern in our system two state-of-the-art methods can be implemented to protect the Beacons against potential attacks: Time-varying IDs [44] and anomaly detection in ID transitions [45]. The first one aims to program the Beacons to broadcast IDs that are not fixed but vary in time and cryptographic techniques such as pseudorandom functions are used to generate such IDs. The downside of this approach is that it may lead to reducing the lifespan of the Beacon’s battery. The second one aims to detect anomalies in Beacon ID transitions by investigating the hypothesis of possible hacked smartwatches. When a user’s smartwatch is moved in the area of the Beacon, the transitions of consecutive Beacon IDs registered by the system should follow a specific probability distribution. The advantage, in this case, is that this method does not require Beacon modification and does not shorten Beacon lifespan.

An Android application was developed and deployed on a Smartwatch with internet capabilities to acquire and process data from the Bluetooth devices in the room and send it to a monitoring database using a messaging system. The Smartwatch watch is worn on the hand of the older adult whose activity we want to detect. The application processes the RSSI signal coming from the Beacons to determine the distance and position using trilateration.

To determine the distance between the Smartwatch from the Bluetooth Beacons installed in a room using the strength of the RSSI signal the following relation was used [46]:

(11) $$d=0.89976\times {\left(P_{R_{x}}/txPower\right)}^{7.7095}+0.111$$

where txPower is established by the device manufacturer and set during its configuration and $P_{R_{x}}$ is the fluctuation of the power of the received signal directly affecting the value of the calculated distance.

**Figure 4:**
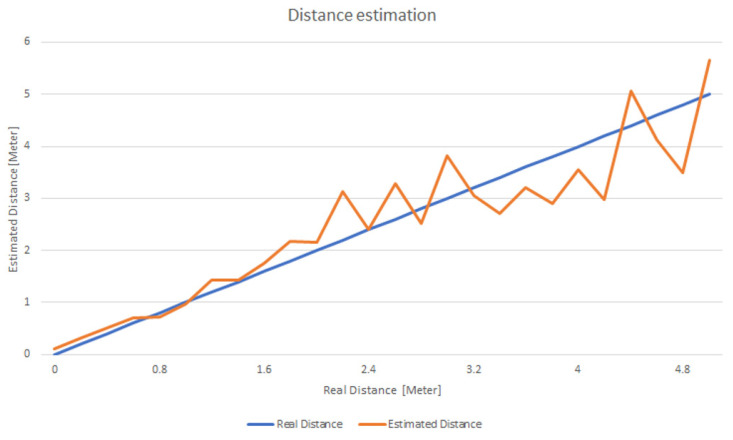
Variation of assessed distance from the Beacon compared with the actual one.

To evaluate the accuracy of the distance determination method, we calculated the distance at a granularity of 0.2 m, gradually moving away from the Beacon (see Figure 4). At each point, data was collected at a granularity of 100 ms for 2 s and the average was calculated. As can be seen the distance estimation works well especially when the distance is smaller than 2 m, making it feasible for use in house rooms. As the distance increases the assessment error also increases but even in this case the average error is below 42 cm.

For daily activity detection minimum three devices are deployed in each room and trilateration techniques are used [47,48] which determine the point of intersection of the three circles generated by the Bluetooth Beacons emitting signals in the room. Table 2 shows the point of intersection (x, y) representing the older adult location inside the room. The central points $\left(x_{1},\;y_{1}\right),\;\left(x_{2},\;y_{2}\right),\;(x_{3},\;y_{3}$) representing the coordinates of the Beacons deployed in the room and circulus radius of the emitted signals $\left(r_{1},\;r_{2},\;r_{3}\right)$ are already known. To ease the calculation and activity identification processes the Beacons are installed in the corners of the room or attached to the objects relevant for the activity carried out (e.g., TV, bed, fridge, book, etc.).

**Figure 5:**
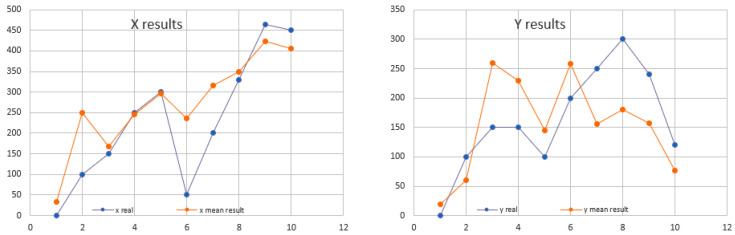
Location results on x and y-axis using trilateration for three Beacons.

The results of using the trilateration technique to determine the location are good, with the average error reported on the *x*-axis being 61.630 cm, and for *y*-axis 69.273 cm (see Figure 5).

The signal strength of the Beacons, together with the calculated distances and coordinates of the location in the house plan, is sent from the Android application to a messaging system and then saved in a non-relational database. This type of message queue offers an asynchronous publish–subscribe mechanism that has the role of maintaining persistent data and ensures high performance and reliability especially in times when the volume of data is very high when the data comes from multiple clients simultaneously, and the database server cannot process them all at the same time.

**Figure 6:**
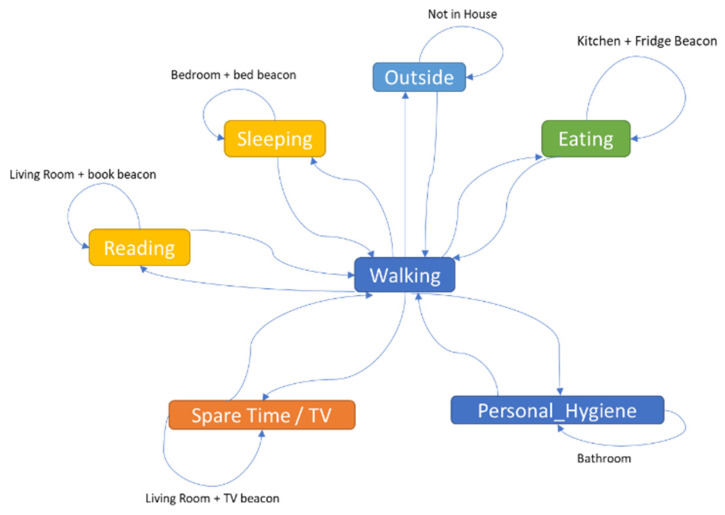
Activity of daily living identification rules.

The data saved in the database were then processed using an inference system to detect the ADLs of the older adult. The daily activities associated with each room of the house and specific objects are (see Figure 6): (i) Sleeping—the older adult is located in the bedroom and then on the bed; (ii) eating—the older adult is in the kitchen and the fridge is used; (iii) personal hygiene—the older adult is in the bathroom; (iv) reading—the older adult is located in the living room and the book is used; (v) spare time/TV—the older adult is in the living room and close to the TV; (vi) walking—the older adult is inside the house and is moving around; (vii) outside—the older adult leaves the house.

### 5.2. Routine and Deviation Assessment

To validate the proposed algorithms for baseline and deviation detection we constructed a data set named ADLS (i.e., the activity of daily living set), which contains information about daily life activities of 10 older adults and was generated using the system described in Section 5.1.

The ADLS data set contains information about daily life activities performed by 10 older adults collected throughout various time frames. We have used it to validate and assess the efficiency of the baseline and deviation detection algorithms in different settings. Table 3 provides a more detailed description of the data including the link to the older adult, which was monitored, the total number of monitored days and out of these the number of days with sequence and duration anomalies.

**Figure 7:**
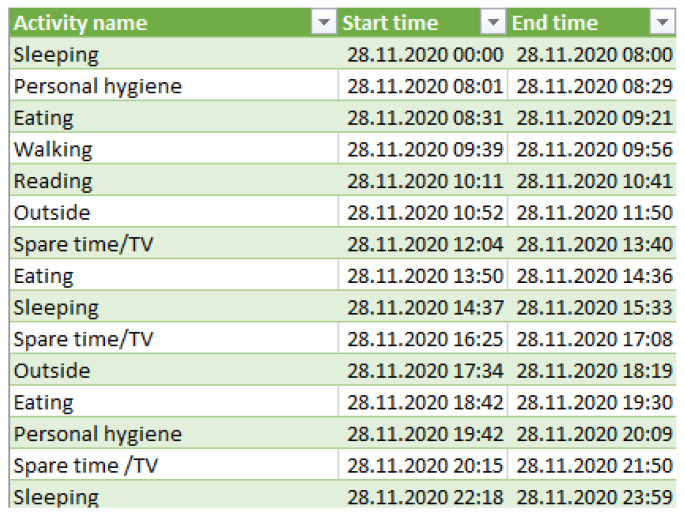
Activities of daily living monitored for a day.

For each older adult, the data set contains the start time and end time of a monitored activity together with the activity label. Figure 7 shows a snippet from the ADLS data set corresponding to the older adult M1, which contains the activities performed during the day of 28 November 2020, together with their start and end time of each activity.

To evaluate the proposed algorithms’ success rate of abnormal behaviour detection, we applied the k-fold cross-validation technique and the results were evaluated using the precision, recall, F-measure, specificity and accuracy metrics. For each older adult, we split the dataset containing the daily monitored activities into k folds, where one fold contains the daily activities monitored for two weeks. For each fold, we performed the following steps:

- The fold was considered as a test data set on which the deviation detection algorithm was applied to detect the days with deviations.
- The remaining folds were considered as part of the training set on which the baseline learning algorithm was applied to identify the routine of the older adult.
- In order to detect deviations, the test set was compared against the identified baseline.
- The results obtained while detecting the deviations in daily life activities were evaluated with the precision, recall, F-measure, specificity and accuracy metrics.

**Figure 8:**
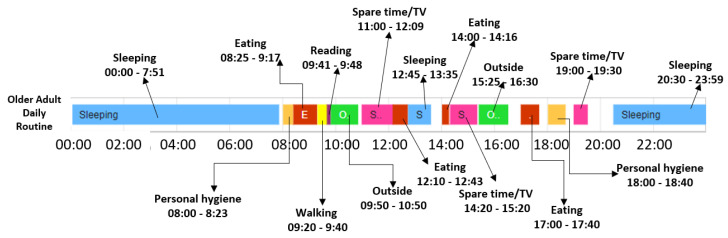
Daily routine extracted for older adult M1.

In what follows, we trace the baseline and deviation detection algorithms considering the k-fold cross validation technique in the case of the older adult M1. The older adult M1 was monitored for 84 days and the corresponding data was split in six folds, where each fold consists of 14 monitored days. Five folds are used to extract the normal behavior, also called the daily routine, while the remaining fold is used to detect the abnormal behavior of the monitored person (i.e., the days that do not follow the daily routine). Figure 8 shows an example of daily routine identified by our algorithm for the older adult M1 considering the start time and end time for each activity, and the duration and chronological order of the activities in the sequence.

Based on the identified baseline and on the days from the five folds considered for baseline detection that are like the baseline in terms of sequence of activities, a confidence interval and a confidence duration were determined for each older adult considered. For example, in the case of the older adult M1, a confidence interval of [0.0794, 0.0829] was determined using Formula (7) while the confidence duration was determined as 0.98.

**Figure 9:**
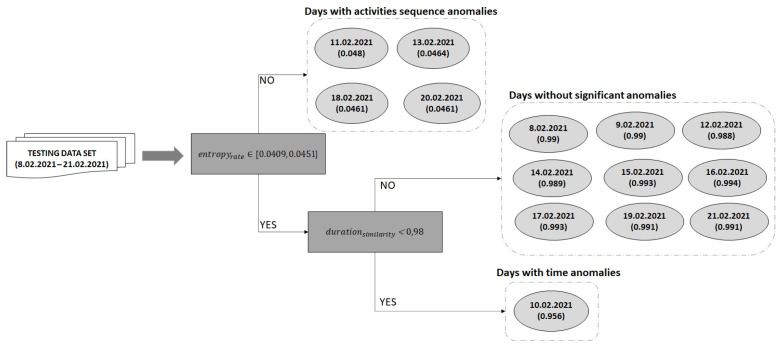
Deviation detection process on the testing data set for older adult M1.

After the daily activities baseline is computed we used the remaining one fold to classify the days that feature deviations from the baseline. The entropy rate of each day from this fold is computed using Formula (6). Figure 9 illustrates the entropy rates computed for all the days in the fold used for detecting deviations for the older adult M1. The days on which the entropy rate does not fall in the confidence interval are classified as days with sequence anomalies.

**Figure 10:**
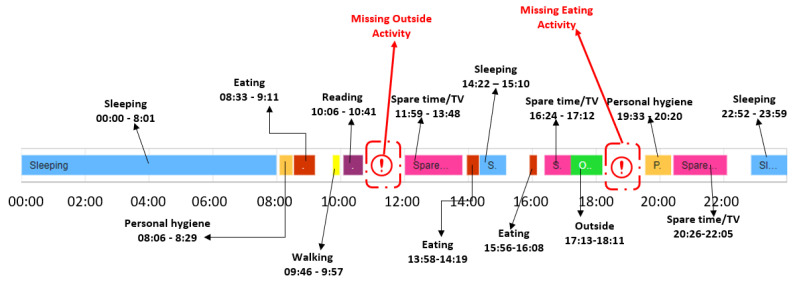
Example of day with activity sequence anomalies for the older adult M1.

Figure 10 illustrates for the older adult M1 an example with the activities from the date 11 February 2021, which has been classified as a day with sequence anomalies.

The days from the one fold that have not been classified as having activity sequence anomalies are further analyzed to detect the ones with activity duration anomalies. For each day a duration similarity is computed which is compared with the confidence duration value (i.e., 0.98). If the duration similarity is lower than the confidence duration, then the day is considered to have time anomalies (see Figure 9).

**Figure 11:**
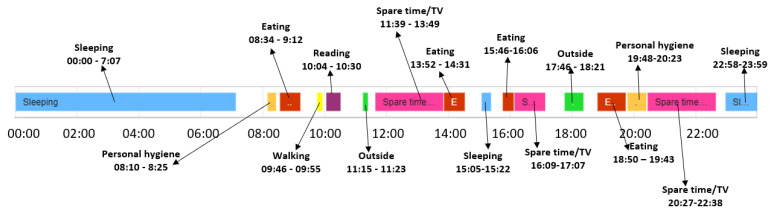
Example of day classified as featuring activity duration anomalies.

Figure 11 illustrates for the older adult M1 the activities from the date 10 February 2021, which has been classified as a day with activity duration anomalies. If the duration similarity is higher than the confidence duration, then the day conforms to the learned baseline, without any significant deviations (e.g., days February 8 and February 9 in Figure 9).

Similarly, we applied the baseline and deviation detection algorithms for all the other folds corresponding to the M1 older adult and for each round of experiments we computed the values of the precision, recall, F-measure, specificity and accuracy metrics.

Finally, Table 4 illustrates the obtained experimental results for each of the 10 older adults of our data set, with the steps followed being the same as the ones presented for older adult M1. The metrics values represent averages of the results obtained when applying the k-fold cross validation technique. The last row in the table presents the overall average values of the considered metrics.

**Figure 12:**
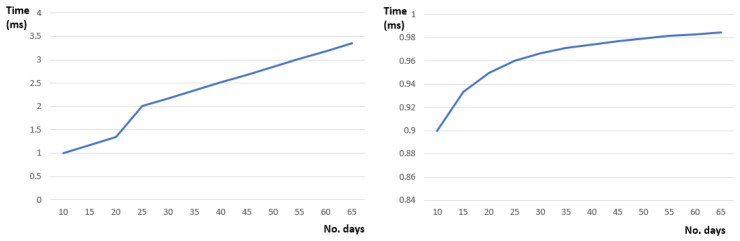
Parameter learning features: (**left**) Learning time and (**right**) probability activity transition (sleep -> eating).

Figure 12 below shows the parameter learning features of our defined Hidden Markov model. Given a sequence of days each having a set of activities the defined model can learn the defined probability parameters with a minimum time overhead (Figure 12—right). Moreover, the learned probability parameter values converge after several iterations throughout consecutive days (see Figure 12—left).

To determine the effectiveness of our approach for anomaly detection in daily life activities we compared the results obtained with the ones reported in the state of the art by [11,17] (see Table 5). As described in the related work section, Refs. [11,17] apply probabilistic models for detecting anomalies in elders’ behaviour. For comparison we considered the values of the precision, recall and F-measure metrics as they were reported by the two approaches. In case of [11] we have averaged the values reported for the above-mentioned metrics considering a sensor activation likelihood of 95% BCI (i.e., Bayesian credible interval) for both real and synthetic data. For [17] we averaged the obtained values for precision, recall and F-measure for various values of the threshold in anomaly detection, a configurable parameter used by authors. By analysing the results from Table 5 it can be noticed that our approach provides better precision than [11,17] with a slight penalty in recall.

## 6. Conclusions

In this paper we proposed a solution for identifying the daily routines of older adults and potential deviations considering the length of the monitored activities and transition probabilities among activities as relevant features. The daily activity monitoring is done using Beacon technology, while for deviation from routine assessment a Markov model is employed. The entropy rate and cosine functions are used to determine the similarity between the sequence of activities registered in a specific day and the routine solution featuring good values of precision. The proposed techniques can identify the daily routines with confidence concerning the activity duration of 0.98 and confidence concerning the sequence of activities in the interval [0.0794, 0.0829]. Compared with other relevant approaches found in the state of the art our solution provides a recall value that is slightly lower and a higher precision value. Related to the baseline learning, the Hidden Markov model shows promising results in terms of determining the activity transition probabilities and learning time being dependent on the amount of data used in training.

## Figures and Tables

**Table 1:** Beacon’s configuration.

Beacon Property		
Advertising Interval	*txPower*	Cal. power 0 m
200 ms	+4 dBm	−21 dBm

**Table 2:** Trilateration technique used for determining the location in a room (adapted from [47,48]).

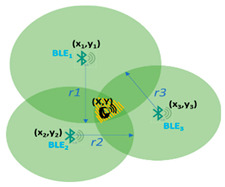	$x=\frac{\left(x_{1}^{2}+y_{1}^{2}+r_{1}^{2}\right)\times \left(y_{3}-y_{2}\right)+\left(x_{2}^{2}+y_{2}^{2}+r_{2}^{2}\right)\times \left(y_{1}-y_{3}\right)+\left(x_{3}^{2}+y_{3}^{2}+r_{3}^{2}\right)\times \left(y_{2}-y_{1}\right)}{2\left(x_{1}\times \left(y_{3}-y_{2}\right)+x_{2}\times \left(y_{1}-y_{3}\right)+x_{3}\times \left(y_{2}-y_{1}\right)\right)}$ $y=\frac{\left(x_{1}^{2}+y_{1}^{2}+r_{1}^{2}\right)\times \left(x_{3}-x_{2}\right)+\left(x_{2}^{2}+y_{2}^{2}+r_{2}^{2}\right)\times \left(x_{1}-x_{3}\right)+\left(x_{3}^{2}+y_{3}^{2}+r_{3}^{2}\right)\times \left(x_{2}-x_{1}\right)}{2\left(y_{1}\times \left(x_{3}-x_{2}\right)+y_{2}\times \left(x_{1}-x_{3}\right)+y_{3}\times \left(x_{2}-x_{1}\right)\right)}$

**Table 3:** ADLS data used in experiments.

Older Adult and Codification	Number of Monitored Days	Number of Days with Sequence Anomalies	Number of Days with Duration Anomalies
M1	84	13	8
W1	42	9	5
W2	112	26	23
W3	70	12	18
M2	84	16	15
W4	42	6	5
W5	56	4	12
M3	98	15	22
M4	56	8	12
M5	70	8	14

**Table 4:** Cross-validation evaluation results.

Older Adult	Precision	Recall	F-Measure	Specificity	Accuracy
M1	0.95	0.86	0.9	0.86	0.85
W1	1	0.73	0.84	1	0.76
W2	1	0.74	0.84	1	0.8
W3	0.88	0.72	0.79	0.83	0.74
M2	0.89	0.81	0.84	0.82	0.8
W4	0.88	0.88	0.87	0.64	0.81
W5	0.98	0.74	0.84	0.75	0.75
M3	0.93	0.79	0.85	0.81	0.78
M4	0.98	0.75	0.84	0.75	0.76
M5	1	0.76	0.86	0.8	0.8
AVERAGE	0.95	0.78	0.85	0.83	0.79

**Table 5:** Comparison results.

SOTA Approach	Precision	Recall	F-Measure
[11]	0.6	0.85	0.63
[17]	0.8085	0.8892	0.7836375
Our approach	0.95	0.78	0.85

## Data Availability

Data sharing is not applicable to this article.
